# Beyond redundant kill: A fundamental explanation of how insecticide mixtures work for resistance management

**DOI:** 10.1002/ps.7180

**Published:** 2022-10-26

**Authors:** Philip G. Madgwick, Ricardo Kanitz

**Affiliations:** ^1^ Syngenta, Jealott′s Hill International Research Centre Bracknell UK; ^2^ Syngenta Crop Protection Basel Switzerland

**Keywords:** population genetics, resistance evolution, mixtures, redundant kill, additional kill, epistasis, modelling

## Abstract

The use of insecticide mixtures for resistance management has been a controversial topic for many decades. Here, we provide a reassessment of the fundamental theory of insecticide mixtures. First, we examine how mixtures differ from other strategies. We suggest that the fundamental strategy concept of a mixture is defined by the simultaneous use of insecticides and their overlapping exposure. Second, we provide a simple, illustrative model to show how mixtures affect resistance evolution. Following the existing literature, we identify a role for ‘redundant kill’ acting against resistant individuals, which we link to the overlapping exposure of insecticides. We also identify the occurrence of ‘additional kill’ acting against susceptible individuals, which is the immediate consequence of the simultaneous use of insecticides. Third, we take a basic approach to the comparison of mixtures and other strategies using a simple model. We find that a common comparison of the time to resistance alone leaves the effects of additional kill unaccounted for. Moreover, we demonstrate that different approaches to comparison can lead to different results because of biases that are introduced in the comparison setup. Fourth, still using the same model, we showcase a more sophisticated approach to comparison using optimised strategies. We find that optimised mixtures always perform better than other strategies due to the combination of redundant and additional kill. We suggest that the comparison of optimised strategies is unbiased because each strategy is performing the best that it can. On this basis, in theory (but not necessarily practice), we believe that mixtures are better than other strategies and, through the steps of our argument, we can tie this success back to the fundamental properties (of simultaneous use and overlapping exposure) that distinguish mixtures from other strategy concepts. © 2022 The Authors. *Pest Management Science* published by John Wiley & Sons Ltd on behalf of Society of Chemical Industry.

## Graphical abstract

We provide a reassessment of the fundamental theory of insecticide mixtures, linking how mixtures differ from other strategies to how mixtures work for resistance management. We suggest that, in theory, mixtures are better than solo‐use strategies for identified reasons.

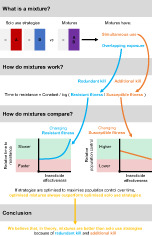

## 1 INTRODUCTION

‘*Of all the strategies proposed to manage resistance, mixtures may be the most controversial*’. (p. 131).^1^

Currently, there are diverse perspectives on the use of insecticides in mixtures for resistance management that tend to be sceptical, which is a consequence of their convoluted history. When the genetic evolution of insecticide resistance first started to receive significant scientific attention with the problems in the mass rollout of DDT after World War II,^2^, ^3^, ^4^ there was interest in how resistance evolution could be better managed or avoided in the future for other insecticides.^5^, ^6^ Studies initially focused on operational factors,^7^ like deploying insecticides more selectively to reduce their overall use and so reduce the selection for resistance.^8^, ^9^, ^10^ These operational countermeasures were expressed in different ways for specific systems of insect control, including reducing the frequency of applications,^11^ dose reduction,^4^ avoiding slow‐release formulations^9^ and so on.^7^ Such approaches came to be understood as common sense acts of ‘moderation’ of insecticide use.^1^, ^11^, ^12^, ^13^ At this time, although mixtures are rarely mentioned directly, the operational principle of moderation implicitly disfavoured the use of mixtures by arguing for less insecticide use.^4^, ^12^

By the 1970s, scientific attention had begun to shift to the impact of genetic and/or biological factors,^14^ which were explored by relying heavily on mathematical modelling using population genetics.^1^ Whilst the tactical manipulation of factors such as immigration,^15^ refugia^14^ and dominance^16^ were studied in their own right,^17^, ^18^ there was growing interest in the promise of the more general strategies of resistance management using multiple insecticides together in rotations (or alternations),^19^, ^20^ mosaics^6^, ^20^ and mixtures (or combinations).^12^, ^21^ In the 1980s, a series of mathematical models based on population genetics sought to disentangle early speculation^4^, ^5^, ^6^, ^12^ on when different approaches to using multiple insecticides would delay the evolution of resistance. Modelling of mosaics and rotations tended to show minimal gains on sequences,^13^, ^22^, ^23^, ^24^, ^25^, ^26^, ^27^ which was often viewed as the ‘nonstrategy’ benchmark^25^, ^28^ where there is a switch in the solo use of insecticides when they fail due to resistance evolution.^22^, ^25^, ^26^, ^27^, ^29^ By contrast, the models of mixtures showed some very substantial delays in resistance evolution in comparison to sequences.^25^, ^28^

From the late 1980s to the early 2010s, these models were increasingly reinterpreted as showing that mixtures only work under restrictive conditions. Indeed, mixtures were argued to need highly effective insecticides with equal persistence, be deployed in the presence of refugia (or untreated sections) and have resistance that is monogenic, recessive and initially rare (or absent).^1^, ^12^, ^13^, ^28^, ^30^, ^31^, ^32^, ^33^, ^34^, ^35^, ^36^, ^37^, ^38^ Alongside parameters that received some analysis in the 1980s models (insecticide effectiveness and recessive resistance), some of the conditions for mixtures to work were taken from assumptions in the setup of those models (monogenic and initially rare resistance), whilst others were never treated with modelling at the time (insecticide persistence and refugia). This extrapolation of when mixtures work was supported by the logic of ‘redundant kill’,^37^ which was taken to provide an explanation of how mixtures work.^34^, ^35^, ^36^, ^39^, ^40^, ^41^ The argument was straightforward: with exposure to a mixture of two insecticides, an individual that is resistant to one insecticide would nonetheless be killed by the other.^28^, ^37^ Strictly, this description assumes that the insecticides have 100% effectiveness, which is an explicit assumption of the model where resistance evolution was first clearly shown to be delayed by orders of magnitude,^25^ and the analysis of other models reported that the utility of mixtures declines rapidly as insecticides become even marginally less effective.^27^, ^28^ Through requiring very high effectiveness and associated factors, the strict logic of redundant kill provided the foundation for a consensus interpretation that the conditions for mixtures to be favoured were too restrictive for mixtures to be useful in reality.^1^, ^27^, ^29^, ^31^, ^35^, ^36^

Recently, a series of new modelling papers have challenged this consensus,^41^, ^42^, ^43^, ^44^, ^45^, ^46^ although these studies have not been framed in these terms. One recent simulation study of insecticides for vector control used massive simulations and a powerful machine learning analysis to address how variation in parameter values contributes toward mixtures being favoured over sequences.^42^ The parameters related to two insecticides included insecticide effectiveness and exposure, and resistance restoration, cost and dominance. The study found that mixtures tended to be favoured by delaying resistance evolution by >20% over sequences across parameter combinations where both insecticides have high effectiveness (>70%) and lower exposures (<60%). An explanation was put forward in a follow‐up study using the same model, which suggested that highly effective insecticides provide protection to each other by killing individuals that are only resistant to one insecticide,^44^ which invokes the same reasoning as put forward by redundant kill.^28^, ^37^ Interestingly, this explanation makes little sense of the ‘secondary’ role of lower exposure, which was left unexplained. A different study, using a very similar model and machine learning approach but running many more simulations, compared two kinds of mixtures to sequences.^43^ Mixtures of the kind from the previous study, with insecticides that have the same effectiveness in mixtures as in sequences, performed similarly to in the previous study. Mixtures that have insecticide effectiveness adjusted to give equal control to sequences were favoured by delaying resistance evolution by >10% over sequences when one or other insecticide has intermediate‐to‐high effectiveness (>35%), whilst other parameters were of much less importance. No explanation was offered for how mixtures work. Another study, running simulations on a model that explicitly incorporated multiple insects*′* life histories, found that mixtures were a robust strategy under high effectiveness, incomplete exposure and a low initial frequency of resistant alleles.^46^ No explanation was put forward, however. A final study explored the optimality of different kinds of mixture in comparison to sequences.^41^ Mixtures with the same dose of each insecticide in mixtures as in sequences were optimal in 52% of simulations, mixtures with half the dose of insecticide in sequences were optimal in 22% of simulations, and sequences were optimal in 20% of simulations. This study invoked redundant kill as the basic explanation of how mixtures work for resistance management.

These recent studies challenge the consensus interpretation of the utility of mixtures by suggesting that mixtures are favoured under broader conditions that are largely defined by both exposure and effectiveness. If we were to suggest where the consensus interpretation deviates, we believe that insufficient attention has been paid to disentangle the conditions where mixtures lead to an *absolutely* longer time to resistance and the conditions where mixtures lead to a *relatively* longer time to resistance in comparison to other strategies like sequences. This becomes apparent in the discussion of the ‘soft fail’ of mixtures (i.e. performing at least as well as sequences), where misunderstandings have arisen around the importance of resistance being recessive and initially rare to the absolute^31^, ^36^ and relative^22^, ^37^ change in the time to resistance, which can lead to a much longer absolute time to resistance for mixtures but little increase in the relative time to resistance in comparison to sequences (see also^42^, ^43^). For discussing when mixtures work by doing something different compared to other strategies, the focus must be on the conditions that lead to a *relative* advantage of mixtures over other strategies.

With such a convoluted history, it can be difficult to make sense of the value of mixtures for resistance management. One empirical way forward is meta‐analysis, to establish what studies generally tend to show about the use of mixtures to delay resistance.^38^ This approach captures the diversity of perspectives, but the ‘wisdom of the crowds’ over the long history of studies can fail to adequately weight the methodological innovations in more recent studies that give them a broader scope. To account for this, a complementary approach is to revisit the theory that supports the use of mixtures for resistance management. We believe, with the promise that mixtures have shown in recent modelling, that the time is ripe for a reassessment of this fundamental theory. We present our argument as simply as we can. First, we differentiate mixtures from other fundamental strategy concepts to formulate a clear definition. Second, we use a simple model of resistance evolution to explain how mixtures work in terms of the differentiating qualities in their definition. Third, we demonstrate the shortcomings of some basic approaches to compare mixtures and other strategies that restrict how mixtures can work. Fourth, we explore a more sophisticated approach to comparison using optimised strategies that impose fewer restrictions. In these four steps, we build the theoretical case, from the foundational definition of mixtures to their perfection in optimisation, that mixtures are better than other strategies for identifiable reasons that fundamentally explain how mixtures work for resistance management.

## 2 WHAT IS A MIXTURE?

**Figure 1:**
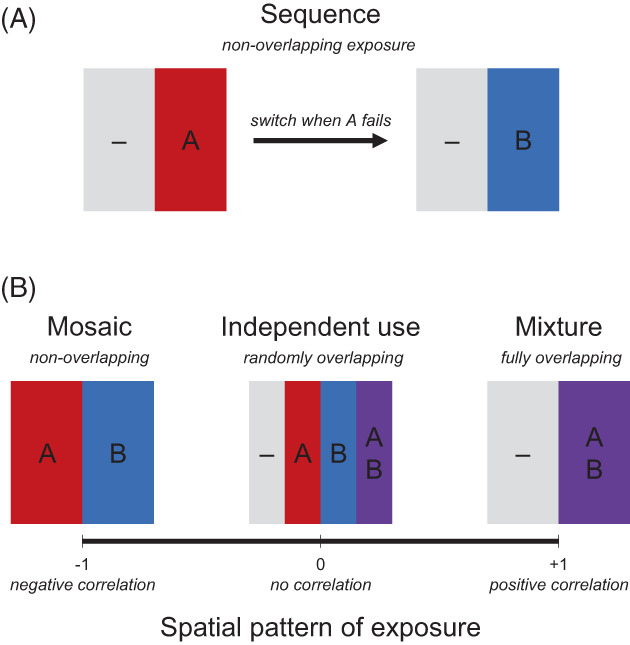
A classification of fundamental strategy concepts for the use of two insecticides based on the pattern of exposure with (A) the solo use of insecticides and (B) the simultaneous use of insecticides. Sequences involve temporally nonoverlapping exposure with the solo use of insecticide A until control failure due to resistance evolution, and then the solo use of insecticide B. Mosaics, independent uses and mixtures involve the simultaneous use of insecticides with different spatial patterns in exposure. Mosaics involve spatially nonoverlapping exposure due to a negatively correlated use of insecticides in different places (e.g. 50% exposure to each insecticide, with 50% exposure to insecticide A or B). Independent use involves randomly overlapping exposure with no correlation in use (e.g. 50% exposure to each insecticide, with 25% no exposure, 25% exposure to insecticide A or B, and 25% exposure to both insecticides A and B). Mixtures involve fully overlapping exposure due to a positively correlated use of insecticides in the same place (e.g. 50% exposure to insecticide, with 50% exposure to insecticides A and B).

It may sound like an obvious question, but establishing what a mixture is provides the first step to understanding the fundamental properties of mixtures that impact resistance management. Moreover, it is important to establish that there is an idealised ‘mixture concept’ that is the subject of theoretical analysis and modelling, which can be different from a practical implementation that is described as a mixture. The mixture concept is set up to represent the most mixture‐like‐mixture, as distinct from other strategy concepts. There are four fundamental strategy concepts^38^: sequences, rotations, mosaics and mixtures. These concepts can be distinguished in the case of the use of two insecticides in a well‐mixed insect population across temporal and spatial dimensions of exposure (Fig. 1).

Sequences involve the solo use of insecticides one at a time (Fig. 1(A)). Although sequences are a solo‐use strategy, they involve the use of multiple insecticides because once resistance has evolved to the first insecticide, there is a switch to the solo use of the second insecticide. Consequently, sequences have nonoverlapping exposure to insecticides because of separate use along a temporal dimension. Rotations, where insecticides are also used solo because of temporal separation, are similar to sequences but involve regularly switching which insecticide is in use. For the purposes of fundamental classification here, rotations are treated as equivalent to sequences based on having a similar temporal separation that ensures a solo‐use pattern of exposure.^22^, ^26^, ^37^

With the simultaneous use of insecticides at the same time, mosaics and mixtures represent extremes along a spatial dimension of the correlation between the exposure to both insecticides. As a point of reference midway between them, it can be helpful to consider the independent use of the two insecticides with uncorrelated treatments that leads to a randomly overlapping pattern of exposure. For example, if there were 50% exposure to each insecticide, then independent use would imply that 25% of the population receives no exposure, 25% of the population receives exposure to one or other insecticide, and 25% of the population receives exposure to both insecticides. Mosaics represent the extreme of negatively correlated treatments in an ‘either‐or’ pattern, so that the exposure to each insecticide never overlaps with the exposure to the other insecticide. Mixtures represent the opposite extreme of positively correlated treatments in an ‘all‐or‐none’ pattern, so that the exposure to insecticides always overlaps.

Overall then, in contrast to other strategies, what distinguishes a mixture? In theoretical terms, a mixture is separated from other fundamental strategy concepts by two properties: the simultaneous use of insecticides and the fully overlapping exposure of those insecticides.

## 3 HOW DO MIXTURES WORK?

To establish how mixtures work, there is necessarily an element of comparison in establishing what mixtures do differently. We believe that the best benchmark for comparison is sequences because they differ from mixtures based on both of the distinguishing properties that we have identified: the simultaneous use of insecticides and the fully overlapping exposure of those insecticides. A simple model of the evolution of resistance to sequences and mixtures is described in Box 1, which is not intended to present new results but rather to illustrate what is already found within existing models.

Strategies are usually compared using the time to resistance.^38^ As Box 1 derived for the spread of an initially rare resistance mutation, the time to resistance in a simple model can be calculated as:

(1) $$\text{Time to resistance}=\text{Constant}/\log\left(\text{Resistant fitness}/\text{Susceptible fitness}\right)$$

**Figure 2:**
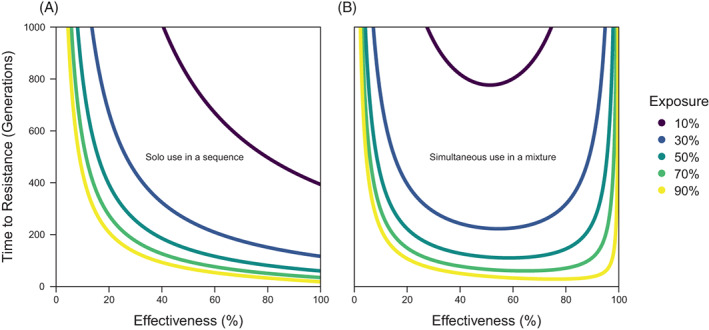
The time to resistance across exposure and effectiveness for (A) sequences and (B) mixtures. The time to resistance is calculated using the simple model in Box 1 in terms of exposure x and effectiveness m, assuming a starting resistant allele frequency of $f_{R,0}=10^{-9}$ and an ending frequency threshold of $f_{R,T}=0.5$. The time to resistance for sequences is $2\log\left(10^{9}-1\right)/\log\left(1/\left(1-\mathrm{xm}\right)\right)$, where the 2 at the start reflects that the time to resistance is calculated for both identical insecticides (that are used one after the other). The time to resistance for mixtures is $\log\left(10^{9}-1\right)/\log(\left(1-\mathrm{xm}\right)/\left(1-x\left(1-{\left(1-m\right)}^{2}\right)\right)$.

The ‘constant’ is the same for all comparisons, and so differences in the time to resistance come down to differences in the fitness ratio of resistant and susceptible individuals. The fitness terms can be calculated based on exposure and effectiveness (Table 1). To make sequences and mixtures comparable, the time to resistance that is calculated for sequences must be doubled to reflect the use of one insecticide after the other, whilst the time to resistance for mixtures is not doubled because insecticides are used simultaneously. The effects of different values of exposure and effectiveness can be plotted (Fig. 2). This reveals that, for sequences, the time to resistance increases with lower exposure and effectiveness in a negative exponential‐like relationship, whereas for mixtures there is a similar relationship except that the time to resistance also increases at higher effectiveness to give a U‐like relationship across effectiveness. Both these relationships are well‐known from previous modelling work.^28^, ^42^, ^43^

**Table 1:** The fitness (ω) of individuals carrying resistant (R) or susceptible (S) alleles under different strategies in terms of exposure (x) and effectiveness (m), where effectiveness is the same for both insecticides

	Sequences	Independent use	Mixtures
$\omega_{S}$	1−xm	${\left(1-\mathrm{xm}\right)}^{2}$	$1-x\left(1-{\left(1-m\right)}^{2}\right)$
$\omega_{R}$	1	1−xm	1−xm
$\omega_{R}/\omega_{S}$	$1/\left(1-\mathrm{xm}\right)$	$1/\left(1-\mathrm{xm}\right)$	$\left(1-\mathrm{xm}\right)/\left(1-x\left(1-{\left(1-m\right)}^{2}\right)\right)$

Box 1 **A simple model**

1 A model is needed for the quantitative comparison of strategies to establish how mixtures work. To make the model as comprehensible as possible to nonmodellers, we have chosen to use the simplest model that demonstrates the fundamental properties of how mixtures work for resistance management. The findings of this simple model are shared with full genetic models that use massive simulations to consider additional properties (e.g. diploidy and dominance, fitness costs, unequal effectiveness etc),^41^, ^42^, ^43^, ^44^, ^45^, ^46^ which anyway found that exposure and effectiveness were the key parameters that distinguished when different strategies were favoured.

Following the model that first clearly articulated the logic of redundant kill^37^ (see also^50^), let us consider the spread of monogenic resistance, meaning that a single resistant allele at independent loci renders each insecticide completely ineffective, against two insecticides in a haploid insect. For simplicity, let us treat the effects of insecticide with just two terms, which have been shown to be critical terms from more complicated modelling^42^, ^43^: the exposure x that describes the proportion of insects that come into contact with each insecticide and the effectiveness m that describes the proportional mortality of a susceptible insect on exposure to each insecticide. To be clear, the effectiveness is assumed to the same for both insecticides.

Using classical population genetics, the selection for resistance between one generation and the next can be calculated in the allele frequency change. For example, the application of an insecticide to an insect population leads to the frequency change of resistant allele R over the susceptible allele S in one generation of:

(B1.1) $$f_{R}^{\prime }=\frac{f_{R}\omega_{R}}{f_{R}\omega_{R}+f_{S}\omega_{S}}$$

where $f_{R}$ is the proportional frequency of the resistant allele R (and $f_{S}=1-f_{R}$ is the proportional frequency of the susceptible allele S), $f_{R}^{\prime }$ is the proportional frequency of resistant allele R in the next generation, $\omega_{R}$ is the fitness of an individual with the resistant allele and $\omega_{S}$ is the fitness of an individual with the susceptible allele. This familiar discrete‐time equation for selection, describing the allele frequency change from one generation to the next, leads to a logistic increase in allele frequency. The discrete‐time equation can be reformulated into a less familiar continuous‐time equation describing the allele frequency change between any number generations, which is a logistic function:

(B1.2) $$f_{R,t}={\left(\frac{\omega_{R}}{\omega_{S}}\right)}^{t}/\left({\left(\frac{\omega_{R}}{\omega_{S}}\right)}^{t}+\frac{f_{S,0}}{f_{R,0}}\right)$$

where 0 is the starting time (i.e. $f_{R,0}$ is the initial frequency of the resistant allele R and $f_{S,0}=1-f_{R,0}$ is the initial frequency of the susceptible allele S) and t is any number of generations into the future. The continuous‐time equation is useful because it permits the calculation of the time it takes for the resistant allele R to reach an arbitrary frequency threshold $f_{R,T}$:

(B1.3) $$T=\log\left(\frac{f_{R,T}\left(1-f_{R,0}\right)}{\left(1-f_{R,T}\right)f_{R,0}}\right)/\log\left(\frac{\omega_{R}}{\omega_{S}}\right)$$

The arbitrary frequency threshold $f_{R,T}$ is chosen to make the time it takes to get there T synonymous with a common‐sense notion of ‘the time to resistance’, which describes when an insecticide fails. Here, as is commonplace,^38^ the time to resistance is taken to be when $f_{R,T}=0.5$, which is the midpoint of the logistic function (and hence its point of symmetry).

Differences between strategies emerge in the interpretation of fitness terms. Here, let us consider three strategies of particular interest: sequences, independent use and mixtures. For a sequence, one insecticide is used until it fails and then the other insecticide is used until it fails. The susceptible fitness is the proportion of individuals that do not die because they are not exposed to the insecticide or do not die on exposure to the insecticide because of incomplete effectiveness: $\omega_{S}=\left(1-x\right)+x\left(1-m\right)=1-\mathrm{xm}$. As resistance renders the insecticide completely ineffective, the resistant fitness is $\omega_{R}=1$.

For independent use, insecticides are used simultaneously with randomly overlapping exposure. The susceptible fitness is the proportion of individuals that do not die because they are not exposed to the insecticide or do not die on exposure to one or other or both of the insecticides because of incomplete effectiveness: $\omega_{S}={\left(1-x\right)}^{2}+x\left(1-m\right)+x\left(1-m\right)+x^{2}{\left(1-m\right)}^{2}={\left(1-\mathrm{xm}\right)}^{2}$. When resistant alleles are at lower frequencies (as they are for the majority of the time from a mutation event until they reach the frequency threshold), if an individual is resistant to one insecticide, it is still susceptible to the other, so $\omega_{R}=\left(1-x\right)+x\left(1-m\right)=1-\mathrm{xm}$.

For a mixture, insecticides are used simultaneously with fully overlapping exposure. The susceptible fitness is the proportion of individuals that do not die because they are not exposed to the insecticide or do not die on exposure to both of the insecticides because of incomplete effectiveness: $\omega_{S}=\left(1-x\right)+x{\left(1-m\right)}^{2}=1-x\left(1-{\left(1-m\right)}^{2}\right)$. Like with independent use, if an individual is resistant to one insecticide, it is still susceptible to the other, so $\omega_{R}=\left(1-x\right)+x\left(1-m\right)=1-\mathrm{xm}$.

For independent use and mixtures when there could be multiple resistant alleles spreading at the same time, the continuous‐time equation that is used to calculate the time to resistance makes a key assumption that there is linkage equilibrium. Strong selection has been suggested to generate linkage disequilibrium that could speed up the time to resistance,^28^ but this has been shown not to be the case.^42^ Moreover, the stochasticity that would be required to generate linkage disequilibrium has also been shown to decrease the probability that resistant alleles spread through a population,^48^ which means that the time to resistance that is calculated for mixtures is likely to be conservative.

How do mixtures work? The classic explanation is redundant kill, where an individual that is resistant to one insecticide would nonetheless be killed by the other.^28^, ^37^ In its original presentation, this logic qualitatively assumed that the insecticides have 100% effectiveness,^25^, ^27^, ^28^, ^37^ which meant that the kill was truly redundant, but the principle can be extended (out of its strict original presentation) to have a quantitative interpretation. Indeed, over the population that encounters a focal insecticide, the redundancy of the kill for any strategy can be calculated as the proportion of the mortality of a susceptible individual that occurs with an individual that is resistant to that insecticide:

(2) $$\text{Redundant kill}\left(\%\right)=100\times \left(\text{Resistant fitness}-1\right)/\left(\text{Susceptible fitness}-1\right)$$

Using Table 1 to describe resistant and susceptible fitness, Eqn (2) calculates that there is zero redundant kill for a sequence and there is $1/\left(2-m\right)$ redundant kill for mixtures. This means that redundant kill is near 50% when the effectiveness (m) is near 0 and near 100% when effectiveness is near 1. There are three important clarifications about redundant kill in here that others have not always made clear. First, some amount of redundant kill is always a property of mixtures, but it is never a property of sequences, nor would it be for any strategy with the solo use of insecticides. Second, for mixtures, the quantity of redundant kill is dependent on effectiveness only (i.e. not exposure), and it increases with higher effectiveness. Third, as redundant kill acts to decrease the resistant fitness in the numerator of the fitness ratio that determines the time to resistance (Eqn 1), redundant kill always increases the time to resistance.

Why does redundant kill occur? Mixtures differ from sequences based on the simultaneous use of insecticides and the overlapping exposure of those insecticides. For mosaics, it is conceivable that redundant kill does not occur with the simultaneous use of insecticides because any one individual in the population is only ever exposed to a single insecticide; insecticides may be used at the same moment in time, but in different places (see Fig. 1). From this extreme possibility of an idealised mosaic, redundant kill increases with more correlated exposure, which is captured in the proportion of the population that is exposed to both insecticides. An idealised mixture represents the opposite extreme to an idealised mosaic where exposure to both insecticides is entirely overlapping. Therefore, whilst redundant kill is enabled by simultaneous use, it differentially arises because of overlapping exposure.

Whilst redundant kill from overlapping exposure is a practical description of what delays resistance evolution, this phenomenon can also be linked into a wider context in population genetics. To consider the meaning of redundant kill, fitness from mixtures can be contrasted against independent use (Table 1). From the independent use of each insecticide with randomly overlapping exposure, the fitness effects of the second insecticide cancel out in the calculation of the fitness ratio at the first resistance locus. Consequently, resistance evolves at the same speed whether an insecticide is being used solo as part of a sequence or simultaneously as part of independent use (as also noted in References 26 and 47), which would mean that the time to resistance for independent use is half that of sequences. So independent use leads to independent evolution. For mixtures, insecticides have fully overlapping exposure, which generates redundant kill that does not cancel out in the calculation of fitness ratios (as also discussed in^48^). Such nonindependent use leads to nonindependent evolution. In classical population genetics, the interaction between the selection of alleles at separate loci is known as epistasis,^49^ but interestingly redundant kill generates this effect because of environmental correlation rather than gene interaction. The extent of the epistatic effect from redundant kill decreases with higher exposures as the difference between the proportion of the population exposed to both insecticides decreases. Indeed, at the extreme of 100% exposure, there is no epistasis as mixtures perform the same as independent use. This link between nonrandom exposure and epistasis, which we believe was first made by,^48^ helps to integrate the study of resistance evolution into the richer theory of population genetics.

As has never been recognised previously (to the best of our knowledge), redundant kill cannot be the entire explanation of how mixtures work because it ignores the immediate effect of the other distinguishing feature of mixtures: simultaneous use. Redundant kill relates to a decrease in the fitness of a resistant individual because whilst that individual is resistant to one insecticide in the mixture, it is not resistant to the other, but the time to resistance depends on the fitness ratio of resistant and susceptible individuals (Eqn 1). In this way, mixtures can also differ from sequences through simultaneous use changing the fitness of a susceptible individual.

As Table 1 describes, susceptible fitness differs for sequences and mixtures. Indeed, for mixtures, the additional use of a second insecticide decreases the fitness of a susceptible individual. All else being equal, this means that mixtures provide a higher level of control of the susceptible population than sequences because the simultaneous use of insecticides kills more susceptible individuals than the solo use of either insecticide. The percentage control of the population prior to the evolution of resistance can be calculated as:

(3) $$\text{Control}\left(\%\right)=100\times \left(1-\text{Susceptible fitness}\right)$$

**Figure 3:**
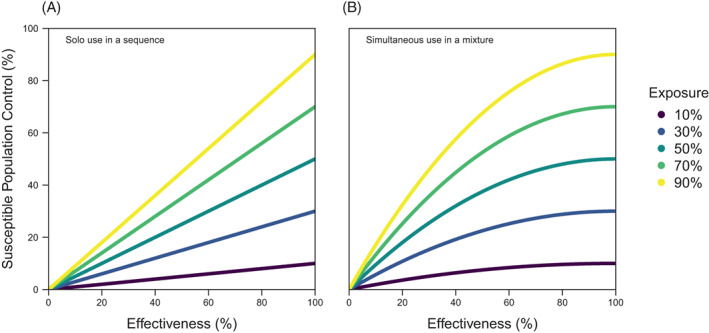
The control of the susceptible population across exposure and effectiveness for (A) sequences and (B) mixtures. The level of control is calculated using Eqn 3, which for sequences is xm and for mixtures is $\mathrm{xm}\left(2-m\right)$, where x is exposure and m is effectiveness.

The level of control varies across exposure and effectiveness (Fig. 3), with higher exposure and effectiveness leading to greater control. The difference between the susceptible population control of mixtures and sequences can be used to define ‘additional kill’. In general terms, additional kill is the change in the proportion of susceptible insects that are killed with the simultaneous use of two insecticides in comparison to the solo use of insecticides. In the simple model, as it typically would be, additional kill is an increase in susceptible mortality. Additional kill for mixtures can be calculated as:

(4) $$\text{Additional kill}\;\left(\%\right)=100\times \frac{\text{Control}:\text{mixtures}\;\text{‐}\;\text{Control}:\text{sequences}}{\text{Control}:\text{sequences}}$$

Using Table 1 to describe susceptible fitness with the use of mixtures and sequences, Eqn (4) calculates that there is zero additional kill when the focal strategy is a sequence and there is $\mathrm{xm}\left(1-m\right)/\left(1-\mathrm{xm}\right)$ additional kill for mixtures. This tells us that the quantity of additional kill is increased by higher exposure (x) and intermediate effectiveness (m). Moreover, in contrast to how redundant kill always increases the time to resistance, as additional kill acts to decrease the susceptible fitness in the denominator of the fitness ratio that determines the time to resistance (Eqn 1), additional kill would always decrease the time to resistance. This is especially interesting under independent use (see Box 1), where the net effect of redundant and additional kill exactly cancels out to half the time to resistance for independent uses against sequences.

As additional kill speeds up the time resistance, does that mean it does not contribute toward explaining how mixtures work? Both redundant kill and additional kill are properties of using insecticides together in mixtures in contrast to using them solo in sequences. Both forms of kill have an effect on the time to resistance, which is the way that strategies have usually been compared in previous studies.^38^ Incorporating additional kill into our understanding of how mixtures work tells us that mixtures can do more than just delay resistance, which we do not believe has previously been formally acknowledged. Mixtures could be used to increase control, which could be desirable in its own right. Alternatively, the formulation of insecticides in a mixture could be used to obtain lower or equivalent control, which could delay the time to resistance. Either way, as has rarely been recognised, both redundant kill and additional kill need to be incorporated into strategy comparison.

## 4 HOW CAN WE SIMPLY COMPARE STRATEGIES?

When making a comparison between strategies, it is important to capture the different kinds of effects that arise from different strategies to ensure that the comparison is ‘fair’. There are multiple possible interpretations of fairness, which complicates matters. Nevertheless, it is important to reflect that this issue was not discussed in the original modelling of mixtures, and still only very rarely receives implicit^41^ or explicit^43^ attention. Going beyond the original modelling's focus on redundant kill, the need to discuss fairness becomes clearer from the identification of additional kill from the simultaneous use of insecticides.

**Figure 4:**
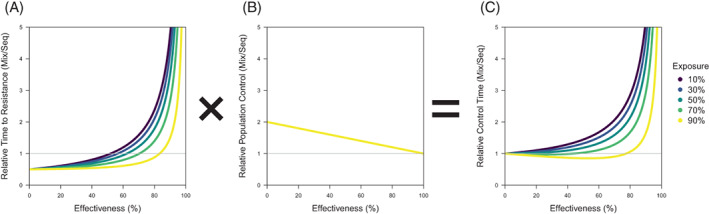
A comparison of mixtures and sequences across exposure and effectiveness in terms of (A) the time to resistance, (B) the control of the susceptible population and (C) control time, whose relationship is depicted by mathematical operators between panels as also shown in Eqn 5. Each metric is given as a relative value, dividing the value for mixtures by the value for sequences, so that a relative value of 1 means that mixtures and sequences are performing equally (given by the grey line). Absolute values of the time to resistance and the control of the susceptible population are given in Figs 2 and 3, respectively. Relative control is the same across exposure. Relative control time is calculated by multiplying the relative time to resistance (in A) by the relative population control (in B).

For two insecticides, a mixture could involve the deployment of both insecticides with the same exposure and effectiveness that they would have under solo use in a sequence, which is the assumption of all the previous studies that have used models to compare strategies that we know of (except^41^, ^43^). Such studies find that mixtures are not a panacea that always delay resistance evolution. Instead, as the simple model that is used here also shows (Fig. 4(A)), sequences can evolve resistance more slowly than mixtures with higher exposure and lower effectiveness. This is explicable in the simple model: as exposure approaches 100% or effectiveness approaches zero, mixtures look increasingly like independent use, and so the contrast is between resistance to the two identical insecticides evolving simultaneously in a mixture or one after the other in a sequence (which would make a mixture take half as long as a sequence). At lower exposure and higher effectiveness, mixtures can perform relatively better than sequences at delaying resistance, which previous studies have concluded is when mixtures work, and this result is often intuitively framed in the logic of redundant kill^27^, ^28^, ^42^, ^44^ (whilst the effect of exposure has been neglected).

The difficulty with the association between when mixtures work and redundant kill alone is that it ignores how additional kill increases the level of population control prior to the evolution of resistance (Fig. 4(B)), which is an unaccounted benefit of using mixtures. One way to incorporate control into the comparison is to shift from comparing the relative time to resistance, to comparing an alternative measure of success.

As this has not been done before (to the best of our knowledge), to provide a simple example to demonstrate such a measure, let us define ‘control time’ as the amount of control that is provided by an insecticide throughout its lifetime of use. Control time incorporates both the level of population control that is achieved, which only occurs for susceptible individuals, and the time that this control is sustained for, which decreases with the evolution to resistance. Control time for a strategy can be estimated as:

(5) Control time=Control×Time to resistance

where ‘control’ and ‘time to resistance’ are defined in Eqns 1 and 3, respectively. Figure 4(C) uses control time to compare strategies, showing the relative control time of mixtures over sequences. This produces a different answer to just comparing the relative time to resistance (Fig. 4(A)), revealing that, under low enough exposure (≤50%), mixtures can always outperform sequences across effectiveness.

Is control time the only fair way to compare strategies? Not at all, because comparing control times is still making a comparison between mixtures and sequences by arbitrarily holding some features constant and allowing others to vary. To be precise, the method of comparing control time in Fig. 4(C) assumes that exposure and effectiveness are constant, which means that strategies vary by how mixtures have overlapping exposure that generates redundant kill and simultaneous use that generates additional kill.

**Figure 5:**
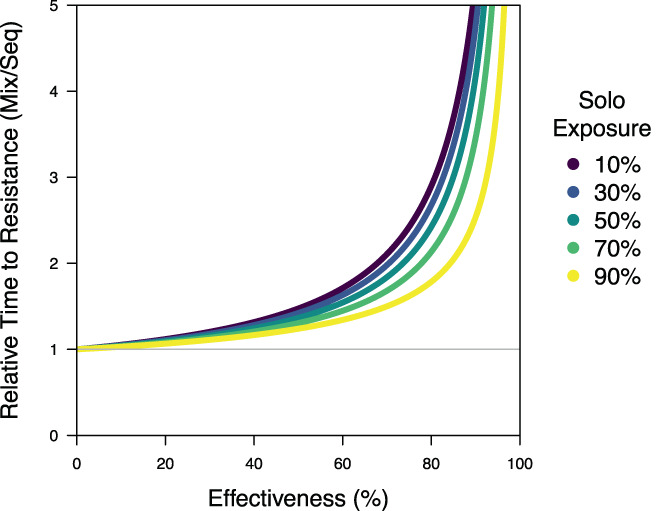
A comparison of the relative time to resistance of mixtures and sequences under equal control, equal effectiveness and unequal exposure. The time to resistance is given as a relative value, dividing the time for mixtures by the time for sequences, so that a relative value of 1 means that mixtures and sequences are performing equally (given by the grey line). The colour legend is given with respect to the exposure of an insecticide when it is used solo in a sequence (x), whilst the exposure of insecticides in mixtures is modified ($x^{\prime }$) to ensure that there is equal control for sequences and mixtures: $1-\mathrm{xm}=1-x^{\prime }\left(1-{\left(1-m\right)}^{2}\right)$ so $x^{\prime }=x/\left(2-m\right)$.

**Figure 6:**
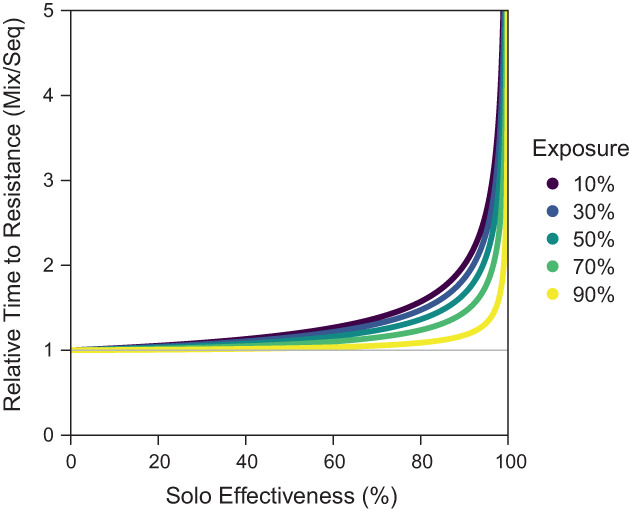
A comparison of the relative time to resistance of mixtures and sequences under equal control, equal exposure and unequal effectiveness. The time to resistance is given as a relative value, dividing the time for mixtures by the time for sequences, so that a relative value of 1 means that mixtures and sequences are performing equally (given by the grey line). The *x* axis is given with respect to the effectiveness of an insecticide when it is used solo in a sequence (m), whilst the effectiveness of insecticides in mixtures are modified ($m^{\prime }$) to ensure that there is equal control for sequences and mixtures: $1-\mathrm{xm}=1-x\left(1-{\left(1-m^{\prime }\right)}^{2}\right)$ so $m^{\prime }=1-\sqrt{1-m}$.

To provide some demonstrations of alternative approaches to ‘fair’ comparison, the time to resistance of strategies can be compared under equal control, meaning that susceptible fitness of mixtures and sequences is held constant and there is no additional kill. In the absence of additional kill, strategy control times only differ based on the time to resistance. In each case, for comparisons under unequal exposure (Fig. 5) or effectiveness (Fig. 6), mixtures always outperform sequences in delaying resistance (in being above the grey line, which describes performing the same as sequences). Therefore, in the absence of additional kill, mixtures always outperform sequences, which must be due to redundant kill. This differs from comparisons under unequal control (Fig. 4), where additional kill can make mixtures perform worse than sequences.

The basic comparisons in Figs 4, 5 and 6, holding some properties constant whilst allowing others to vary, show that results depend on how mixtures are compared to sequences. Such basic comparisons are plagued by introduced bias because there is no straightforward way to independently justify a notion of ‘fair comparison’ without imposing constraints on how mixtures are permitted to work, which at least emphasises the importance of explicitly addressing this question. For most practical purposes, a useful approach would be to pick the notion of fairness that captures how mixtures can be designed within the real‐world constraints of the system under investigation. For example, it could be that a product would need to meet a minimal level of control to be used in preference over competing products, which could favour comparisons at a minimum performance threshold. However, the challenge in settling for a practical resolution that accepts multiple notions of fairness is that the scope of analysis is shifted away from addressing the fundamental question about how, in theory, mixtures compare to other strategies.

Box 2 **Basic comparisons**

For the use of two insecticides, ‘fair’ comparisons are made by finding a sequence that has a logical relationship to a mixture. Initially in the main text, the relationship was assumed to be on the basis of the insecticides having the same effectiveness and exposure in sequences and mixtures. This generates additional kill because the simultaneous use of two insecticides increases control. This should be incorporated into comparison, for example by comparing control time (Fig. 4(C)). Other fair comparisons can be made by comparing sequences and mixtures under equal control, allowing either exposure or effectiveness to vary in mixtures in comparisons with sequences (whilst also holding either effectiveness or exposure equal, respectively). As control is equal, the comparison can focus on the relative time to resistance only, which would be the same as comparing relative control time.

Comparisons under equal control involve calculating a new exposure or effectiveness term, depending on which is allowed to vary. The new term that is used in the calculation of susceptible and resistant fitness for mixtures can be solved by finding when the susceptible fitness of sequences and mixtures is the same (see Table): $1-\mathrm{xm}=1-x\left(1-{\left(1-m\right)}^{2}\right)$. First, if exposure is unequal between mixtures and sequences, the exposure to both insecticides that affords equal control under equal effectiveness is $x^{\prime }=x/\left(2-m\right)$. Second, if effectiveness is unequal between mixtures and sequences, the effectiveness of both insecticides that affords equal control under equal exposure is $m^{\prime }=1-\sqrt{1-m}$. Table 2 describes the effect that using these new exposure and effectiveness terms has on fitness.

**Table 2:** The fitness (ω) of individuals carrying resistant (R) or susceptible (S) alleles under different mixture treatments in terms of exposure (x) and effectiveness (m), where control (or susceptible fitness $\omega_{S}$) is the same for both sequences and mixtures (cf. Table 1)

	Mixture, unequal exposure	Mixtures, unequal effectiveness
$\omega_{S}$	1−xm	1−xm
$\omega_{R}$	$1-\left(x/\left(2‐m\right)\right)m$	$1-x\left(1-\sqrt{1-m}\right)$

## 5 HOW CAN WE BETTER COMPARE STRATEGIES?

A bold, new way forward to compare strategies in a more flexible way is to contrast optimised strategies. At present, even recent modelling has only gone so far to compare selected possibilities of how a mixture can be formulated (e.g. solo‐, half‐ and reduced‐dose formulations).^41^, ^43^ There is a broader scope for optimisation to permit all possible formulations to be considered in an unbiased way. Optimisation requires defining what makes a strategy successful, which must include all the dimensions of success to ensure that they are reconciled in the optimal solution. Using the simple model from Box 2 and control time as the quantity to be maximised (Eqn. 5), an optimisation process can be demonstrated.

**Figure 7:**
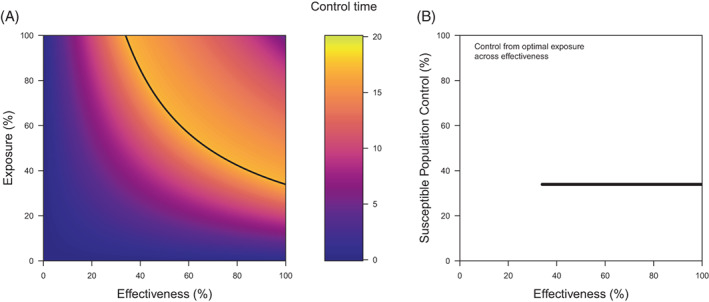
An optimisation of sequences by control time for exposure across effectiveness in terms of (A) the optimality surf and (B) the effect of the optimal solutions on the control of the susceptible population. In (A), the black line describes the optimal solutions for a timeframe of 100 generations. In (B), control is not evaluated below a threshold effectiveness, which corresponds to when the optimal solutions would demand an exposure that is greater than 100% (which is impossible).

**Figure 8:**
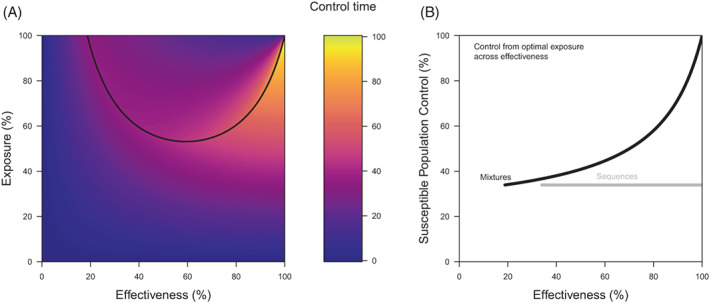
An optimisation of mixtures by control time for exposure across effectiveness in terms of (A) the optimality surface and (B) the effect of the optimal solutions on the control of the susceptible population. In (A), the black line describes the optimal solutions for a timeframe of 100 generations. In (B), control is not evaluated below a threshold effectiveness, which corresponds to when the optimal solutions would demand an exposure that is greater than 100% (which is impossible). Also in (B), the optimal solutions in terms of control are evaluated for mixtures (in black) and sequences (in grey) for comparison.

As Box 3 describes in greater detail, a straightforward optimisation to maximise control time on its own favours a minimal level of control over a maximal time to resistance. If, to make the result more practical, the optimisation is tweaked to maximise control time within a timeframe of interest (e.g. 100 generations), a set of optimal solutions for exposure can be calculated across effectiveness for sequences (Fig. 7) and mixtures (Fig. 8). For sequences, an interesting property of the optimal solutions (the black line in Fig. 7(A)) is that control is equal across all combinations of optimal exposure and effectiveness (Fig. 7(B)). In contrast to sequences, optimal solutions for mixtures (the black line in Fig. 8(A)) lead to increasing control with higher effectiveness (the black line in Fig. 8(B)).

Box 3 **Comparing optimised strategies**

Basic comparisons, involving holding some factors constant whilst allowing others to vary, need to explicitly consider fairness, which can be circumvented by contrasting optimised strategies. Instead of directly specifying what factors should be held constant, optimisation relies on a criterion of success that indirectly specifies how important different factors are. Here, with respect to resistance management, control time is taken as the quantity to be maximised, which is a logical choice because it describes the amount of control that is provided by an insecticide over its lifetime of use.

To start with, consider a straightforward optimisation where control time is maximised without any other constraints. Control time is calculated as the level of susceptible population control multiplied by the time to resistance. The maximum control time for each strategy across exposure (x) and effectiveness (m) should be able to be found by calculus. However, a difficulty in using calculus arises in that the log fitness ratio makes it impossible to separate terms. Instead, if control time is numerically evaluated (across 0<x<1 and 0<m<1), we find that the maximum control time for sequences occurs as effectiveness approaches 0 and exposure approaches 0, and for mixtures occurs as effectiveness approaches 1 and exposure approaches 0. The shared property of exposure approaching zero means that control time is maximised by using a minimal level of control over a maximal period of time. For mixtures, the level of control can be higher than for sequences primarily because of redundant kill. The shared result is the consequence of the time to resistance increasing faster than the control of the susceptible populations decreases, which is unsurprising when it is recalled that the time to resistance has no upper bound (i.e. the time to resistance can tend towards positive infinity, whilst the population control tends toward a lower bound of zero).

This straightforward optimisation of control time produces a solution that would be practically unacceptable. Although it is impractical, it affords important two findings. First, additional specifications beyond ‘maximising control time’ are needed make the optimisation calculate a more practical solution. Second, following the pattern of results, if those specifications detailed a minimum standard of control alone, we should expect that the optimal solution would be to minimally meet that standard of control to give a maximal time to resistance, which will be longer for mixtures than sequences (following the basic comparison under equal control in Fig. 6).

To go beyond a trivial result, a more practical optimisation using the simple model can be conducted by specifying that the maximisation of control time should take place within a timeframe of interest. The timeframe of interest may be defined by, for instance, the time it takes to develop a new product. By way of example, the timeframe of interest is set to 100 generations, which may be interpreted as 10 years with 10 insect generations per year. Accepting that there may also be constraints on the exposure and effectiveness that can be practically achieved, the combination of exposure and effectiveness that maximises control time can be calculated for exposure as a function of effectiveness. For both sequences and mixtures, the optimal exposure ($\hat{x}$) for any value of effectiveness is solved by finding the combination of values that leads to a time to resistance that equals the timeframe of interest (T=100). For sequences, the optimal exposure is calculated as:

(B3.1) $${\hat{x}}_{\mathrm{SEQ}}=\left({\left(\frac{f_{R,T}\left(1-f_{R,0}\right)}{\left(1-f_{R,T}\right)f_{R,0}}\right)}^{\frac{2}{T}}-1\right)/\left(m{\left(\frac{f_{R,T}\left(1-f_{R,0}\right)}{\left(1-f_{R,T}\right)f_{R,0}}\right)}^{\frac{2}{T}}\right)$$

An interesting property of this solution (the black line in Fig. 7(A)) is that control, which is calculated following Eqn (5), is equal across all combinations of optimal exposure and effectiveness (Fig. 7(B)). For mixtures, the optimal exposure is:

(B3.2) $${\hat{x}}_{\mathrm{MIX}}=\left(1-{\left(\frac{f_{R,T}\left(1-f_{R,0}\right)}{\left(1-f_{R,T}\right)f_{R,0}}\right)}^{\frac{1}{T}}\right)/\left(m\left(1-\left(2-m\right){\left(\frac{f_{R,T}\left(1-f_{R,0}\right)}{\left(1-f_{R,T}\right)f_{R,0}}\right)}^{\frac{1}{T}}\right)\right)$$

In contrast to sequences, optimal exposure for mixtures (the black line in Fig. 8(A)) leads to increasing control with higher effectiveness (the black line in Fig. 8(B)).

How do optimal sequences and mixtures compare to one another? Both optimal solutions lead to resistance evolving at the same time, which is at the end of the timeframe of interest (after 100 generations for Figs 7 and 8). Consequently, control time only differs between strategies based on the level of control that each optimal strategy provides. All the levels of control provided by mixtures across effectiveness exceed the level of control provided by sequences (Fig. 8(B)). For this reason, we would argue that, in their fundamental comparison, mixtures are better than sequences. Why is this the case? Higher control arises from additional kill, but the attainment of higher control without changing the time to resistance is due to redundant kill; the separation of the contributions of these two effects is difficult because their success really comes from their interaction.

If different criteria of success were used, could sequences be better than mixtures? Yes, absolutely. One important and unaccounted consideration may well be that the simultaneous use of two insecticides could have a greater economic cost than the solo use of one insecticide (see also^10^, ^13^). Deciding whether or not the greater cost of a mixture product outweighs any benefits in control time would ultimately come down to, for instance, the assessment of how the control of pest damage impacts the return from crop yield. At the very least, this would require incorporating economic factors into the criterion of success, which would undoubtedly require real‐world parameterisation. Whilst this would be highly informative, we believe that the optimisation using the simple model that is used here, which is sufficiently complicated to distinguish fundamental strategy concepts, provides a more fundamental assessment of optimised mixtures.

## 6 CONCLUSION

In theory, are mixtures fundamentally better or worse than sequences? We believe that mixtures are better than sequences. We would support this claim with appeal to the fundamental description of what a mixture is, how they work and how this shapes their comparison. We identified that a mixture differs from other strategies through both the simultaneous use and overlapping exposure of insecticides. We showed that overlapping exposure generates redundant kill that delays resistance evolution because an individual that is resistant to one insecticide can be killed by the other, and simultaneous use generates additional kill wherein the use of insecticides together kills more susceptible individuals. We examined that there are multiple ways to compare mixtures to sequences. Basic approaches, which hold some features the same as other strategies whilst allowing others to vary, can lead to different results. Yet, if we compare optimised strategies, we find that mixtures always outperform sequences, which we can explain in terms of redundant and additional kill that arise because of overlapping exposure and simultaneous use, respectively. Does this mean that a mixture is always going to outperform a sequence in practice? Of course not. First, the theoretical comparison is based on optimised strategies, and there is no reason to think that real examples of mixtures are close to their optimal formulations for resistance management (when this is not currently a driver in their design). Second, there may well be practical constraints or additional considerations that have not entered into the theoretical comparison. Nonetheless, the triumph of mixtures in theoretical comparisons should act as a guide for future work on their practical deployment.

## 7 FUTURE DIRECTIONS

We believe that the trajectory of future research into insecticide mixtures must address the history of scepticism. When mixtures were first proposed, mixtures were initially disfavoured because the implied use of more insecticide acts against the operational principle of moderation. Modelling found situations when mixtures could be useful for resistance management, but these results were subsequently reinterpreted to suggest that the conditions that favour mixtures could rarely occur in reality. We believe this established a long‐reigning consensus that still leads many people to think that insecticide mixtures are not desirable for resistance management.^1^, ^27^, ^29^, ^31^, ^35^, ^36^ Here, we have argued against this perspective by decomposing why mixtures are better than other strategies in a simple model back into the fundamental properties that distinguish mixtures from other strategies. Of course, there are many additional considerations that we have not discussed, but we believe that our simple argument would hold regardless of such complications as a step towards challenging the consensus. However, to convincingly establish a new consensus, three lines of evidence would need to be assembled.

First, there would need to be the experimental testing of the benefits of mixtures. This has been a longstanding suggestion,^12^, ^13^, ^17^, ^28^, ^30^, ^36^, ^39^ albeit that the consensus implied that this would not be a fruitful area of research^12^, ^13^, ^36^ and so it has been neglected. Ideally, experimental testing should consider the effects of stochasticity on evolution in large pest populations, which may require innovative experimental design. Second, the theoretical description of the differential effects of mixtures would need to be bolstered to explain the phenomena in experimental data. Recent modelling has made some progress,^41^, ^42^, ^43^, ^44^, ^45^, ^46^ but the comparisons between mixtures and other strategies have tended to be simplistic and, for example, leave the benefits of additional kill unaccounted for. Here, the study of insecticide mixtures could benefit from comparisons with the parallel literature on fungicide mixtures,^47^, ^51^, ^52^, ^53^ which are commonly used for resistance management. Third, mixtures would have to be evaluated practically, and the best way to do this would be to attempt to develop mixture products following design principles from resistance management. One important consideration may well be that mixture products could be more expensive than the solo products,^10^, ^13^ which could lead to predictable compromises in their design (like lower doses) that could be brought into theoretical comparisons. Renewed interest in the development of mixtures may also refocus attention on the regulatory obstacles that still exist in bringing mixture products to market.^43^, ^54^ Therefore, having revisited the fundamental explanation of how insecticide mixtures work for resistance management to reset the theory of mixtures on a firmer foundation, we believe that the time is ripe for renewed scientific interest in the multifaceted challenges of deploying mixtures to translate the promise of recent theoretical studies into real‐world successes.

## Data Availability

Data sharing not applicable to this article as no datasets were generated or analysed during the current study.
